# Supplementary material for: Molecular and phenotypic analysis of rodent models reveals conserved and species-specific modulators of human sarcopenia
Source: Commun Biol. 2021 Feb 12;4:194. doi: 10.1038/s42003-021-01723-z (PMC7881157; doi:10.1038/s42003-021-01723-z)
Supplement: Supplementary file 2 — Supplementary Information [file 42003_2021_1723_MOESM2_ESM.pdf]

Supplementary Fig. 1-13

Molecular and phenotypic analysis of rodent models reveals conserved and species-specific modulators of human sarcopenia

Anastasiya Börsch[#1], Daniel J. Ham[#2], Nitish Mittal[1], Lionel A. Tintignac[3], Eugenia Migliavacca[4], Jérôme N. Feige[4], Markus A. Rüegg[*2], Mihaela Zavolan[*$1]

[#]These authors contributed equally
[*]These authors jointly supervised this work
[$]Corresponding author (mihaela.zavolan@unibas.ch).

Affiliations

1. Biozentrum, University of Basel and Swiss Institute of Bioinformatics, CH-4056 Basel, Switzerland;
2. Biozentrum, University of Basel, CH-4056 Basel, Switzerland;
3. Department of Biomedicine, Pharmazentrum, University of Basel, CH-4056 Basel, Switzerland;
4. Nestlé Research, EPFL Innovation Park, CH-1015 Lausanne, Switzerland.

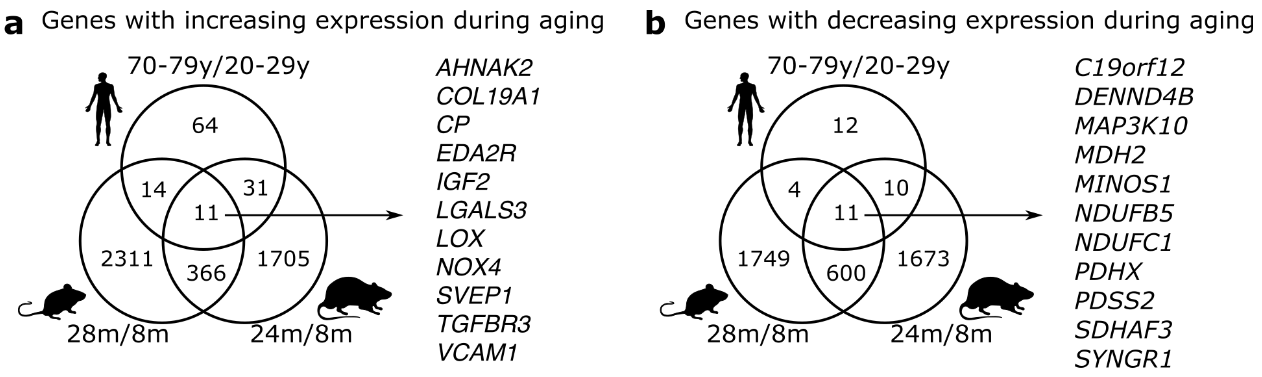

Supplementary Fig. 1. Venn diagram of differentially expressed genes in gastrocnemius muscles of old compared to young individuals of the three species. **a** Genes with significantly increased expression at high age. **b** Genes with significantly decreased expression at high age. Differential expression analysis was performed with the EdgeR tool[31]. FDR<0.01 was considered as the significance threshold. Only genes with orthologs across all studied species were considered.

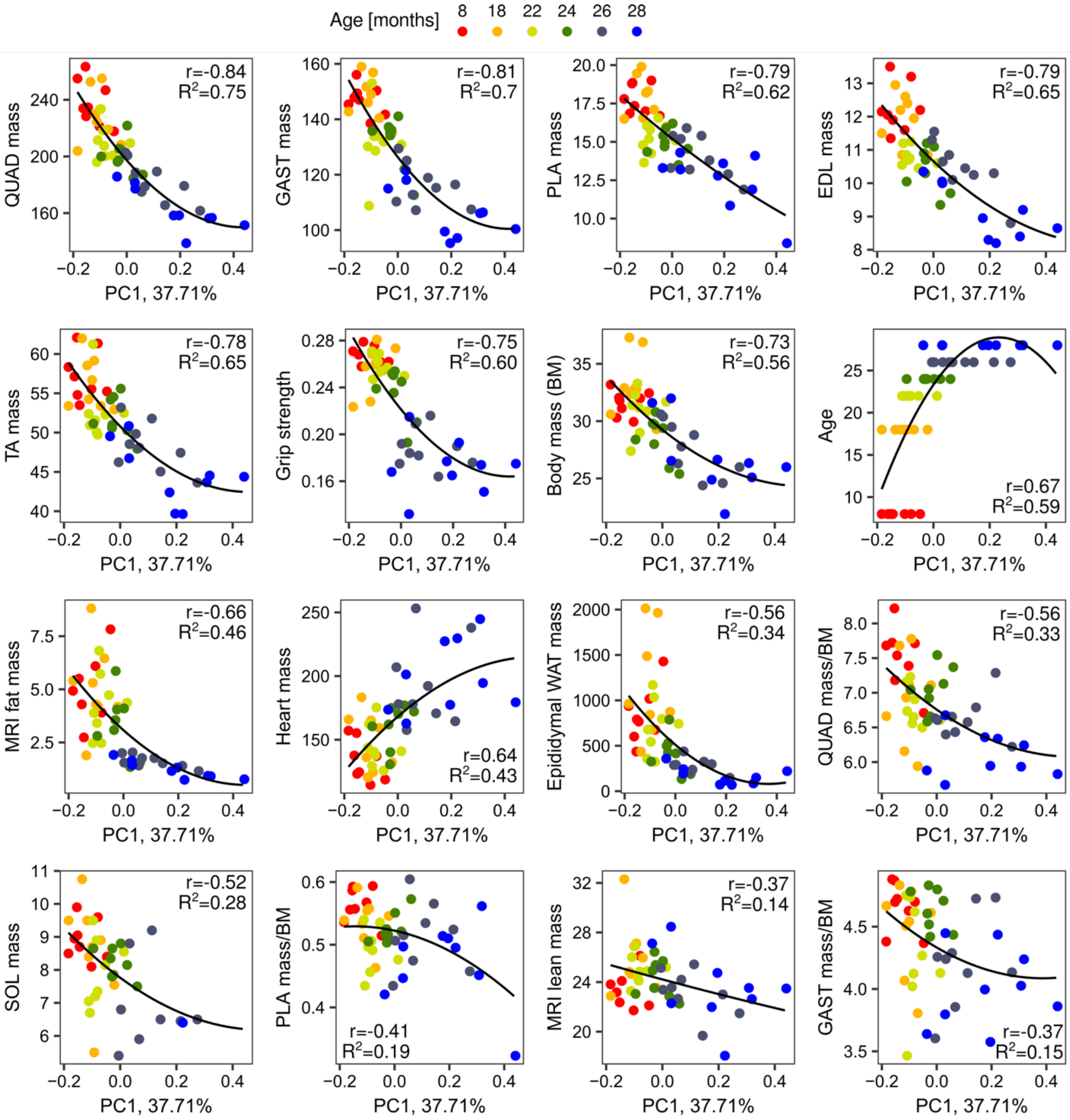

Supplementary Fig. 2. Scatter plots of PC1 coordinates and phenotypic measurement values for mouse samples. Only the 16 phenotypic parameters with the highest absolute correlation with PC1 are shown. Black curves correspond to 2nd order polynomial fits to the data. ‘r’ is the Pearson correlation coefficient, R^2 is the coefficient of determination.

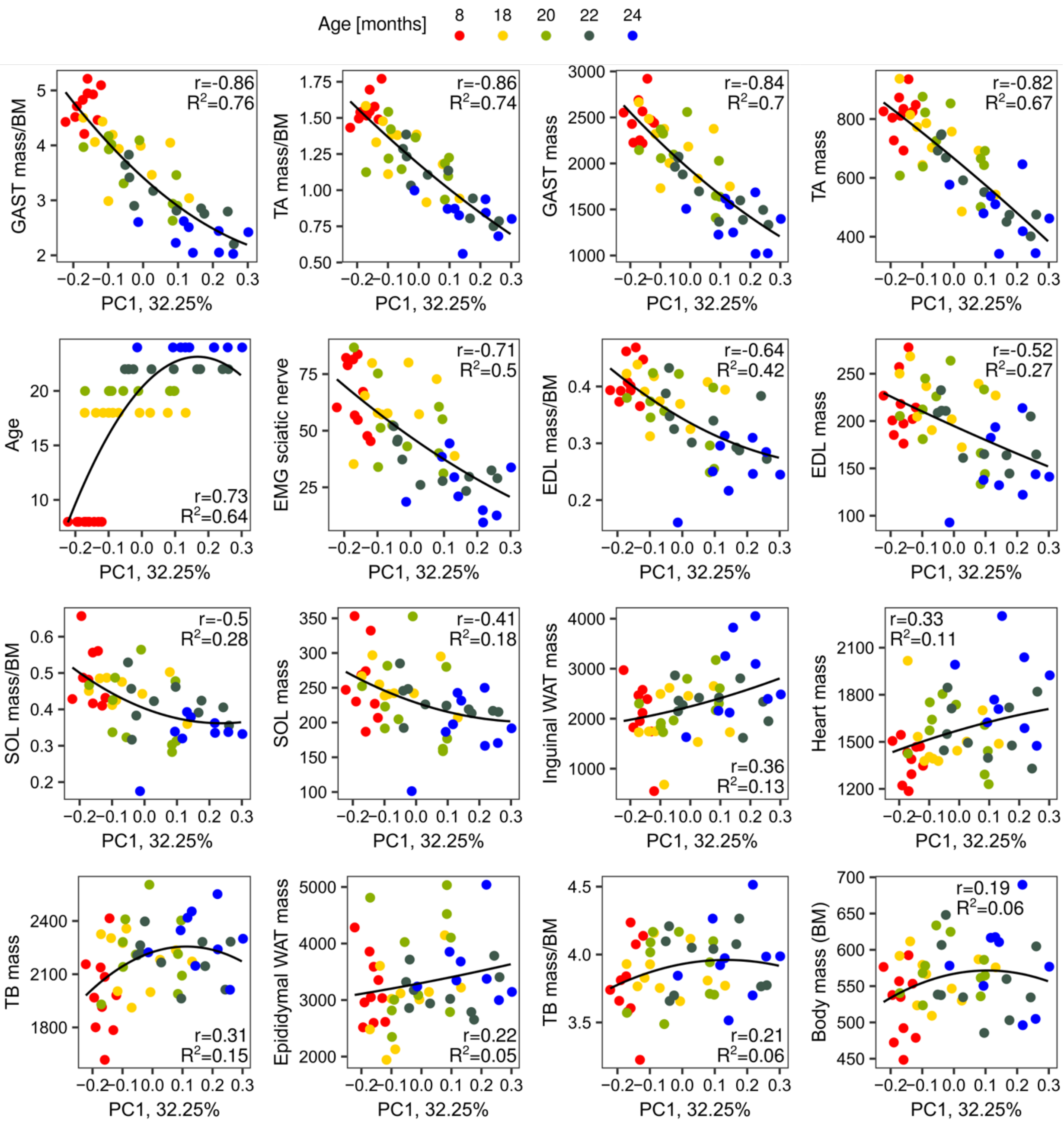

Supplementary Fig. 3. Scatter plots of PC1 coordinates and phenotypic measurements for rat samples. Only the 16 phenotypic parameters with the highest absolute correlation with PC1 are shown. Black curves correspond to 2nd order polynomial fits to the data. 'r' is the Pearson correlation coefficient, R^2 is the coefficient of determination.

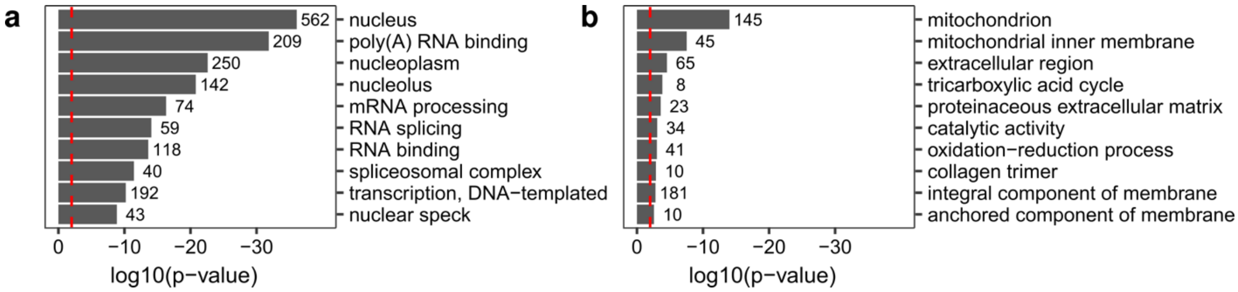

Supplementary Fig. 4. Gene ontology (GO) analysis of genes with significantly different expression levels in groups ‘G1’ and ‘G2’ (Fig. 3b, dashed ellipses). **a** Top 10 GO terms for genes with significantly higher expression levels in replicates from ‘G2’ in comparison to replicates from ‘G1’. **b** Top 10 GO terms for genes with significantly lower expression levels in replicates from ‘G2’ in comparison to replicates from ‘G1’. GO analysis was performed in DAVID[87]. Red dashed lines designate the significance threshold p-value<0.01. Numbers next to bars designate the number of genes that enriched the GO term.

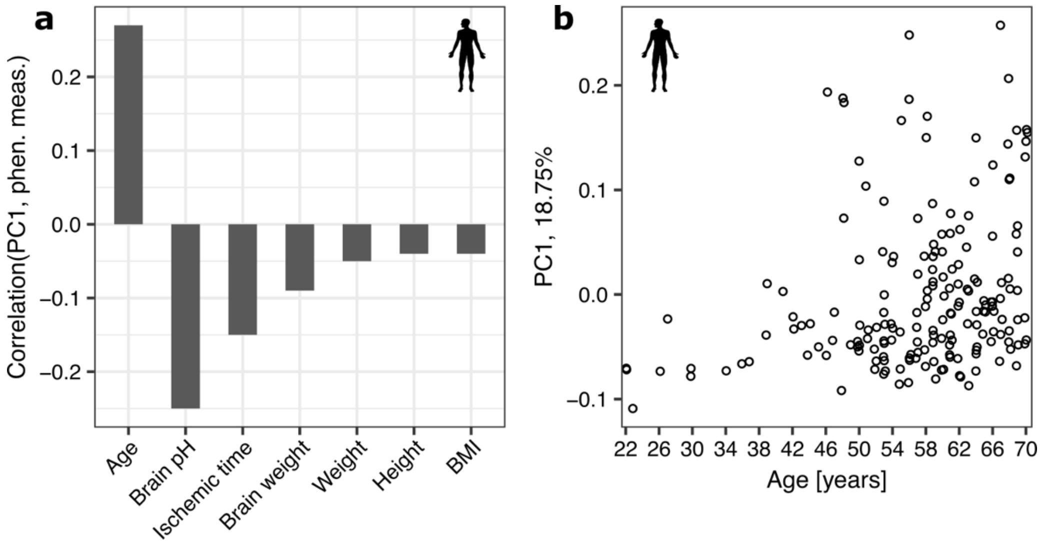

Supplementary Fig. 5. Correlation between PC1 of gastrocnemius muscle transcriptome data and phenotypic measurements in humans. A publicly available RNA-Seq data set for individuals aged between 22 and 70 years, available through the GTEx project, was utilized[24]. **a** Values of the Pearson correlation between PC1 and selected phenotypic measurements. Abbreviations: BMI- body mass index. **b** PC1 coordinates for the RNA-Seq data set collected for human gastrocnemius muscles.

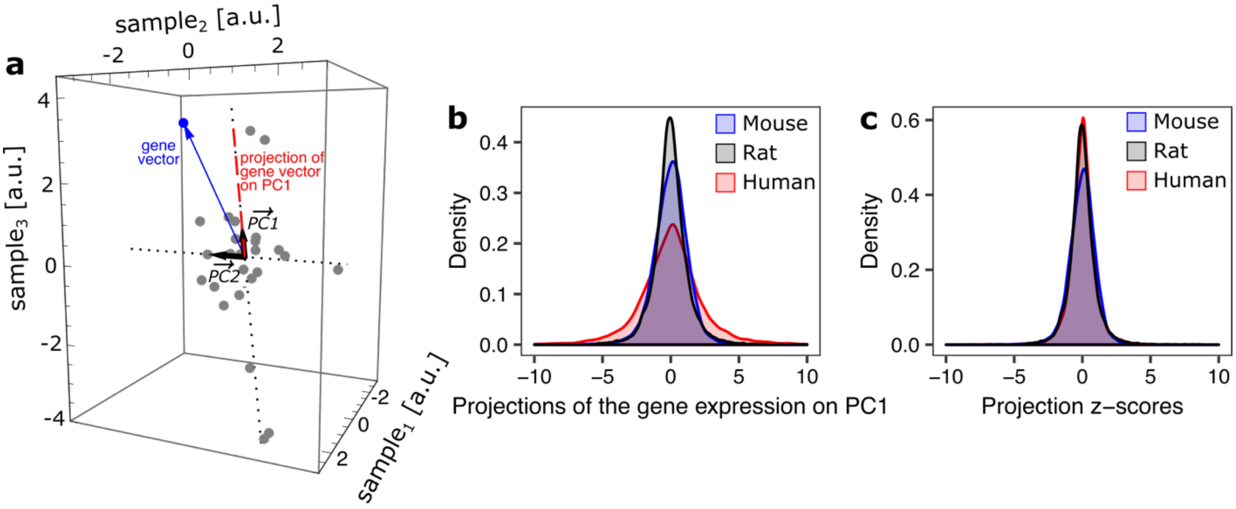

Supplementary Fig. 6. Calculating projections of the gene expression on PC1. **a** Visualization of gene expression in the sample space. Each dot corresponds to a gene. Gene coordinates correspond to expression levels in individual samples, after subtraction of the mean across samples. Black vectors designate PC1 and PC2 of the data set. Each gene can be associated with a vector starting from the origin and aiming at the point corresponding to the gene coordinates in the sample space (blue vector). Red dashed line indicates the projection of a representative gene vector (blue vector) on PC1 (black vector). **b** Distribution of projection values of gene expression vectors on the corresponding PC1 across species. **c** Distribution of projection z-scores across species.

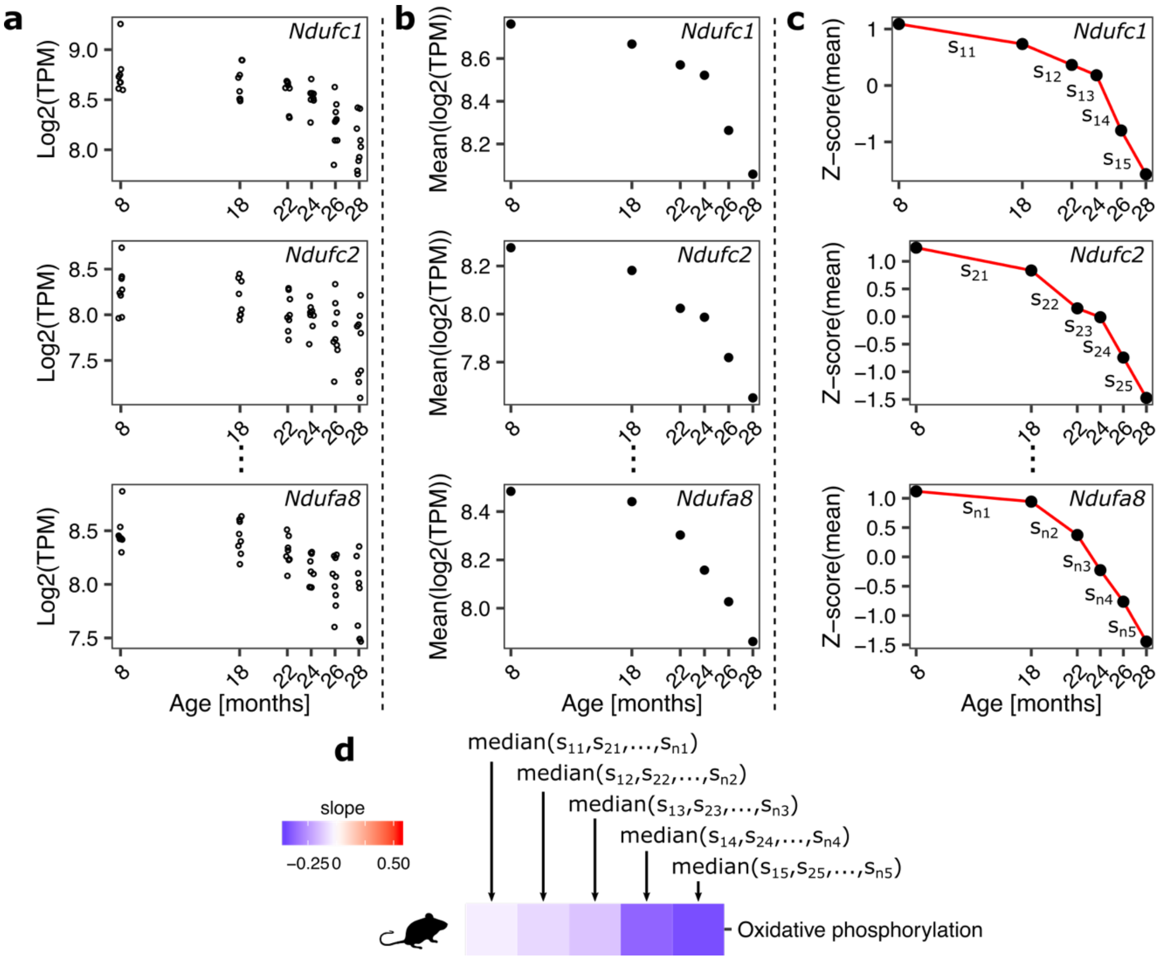

Supplementary Fig. 7. Calculating slopes for age-related changes in the expression of genes forming the leading edge of the KEGG pathway 'Oxidative phosphorylation' for the mouse data set. **a** Expression of leading-edge genes during aging. **b** Mean expression in replicates of the same age. **c** Z-scores of the mean expression. The slopes of z-score changes for neighboring time points were calculated. **d** Median values of slopes across genes from the leading edge.

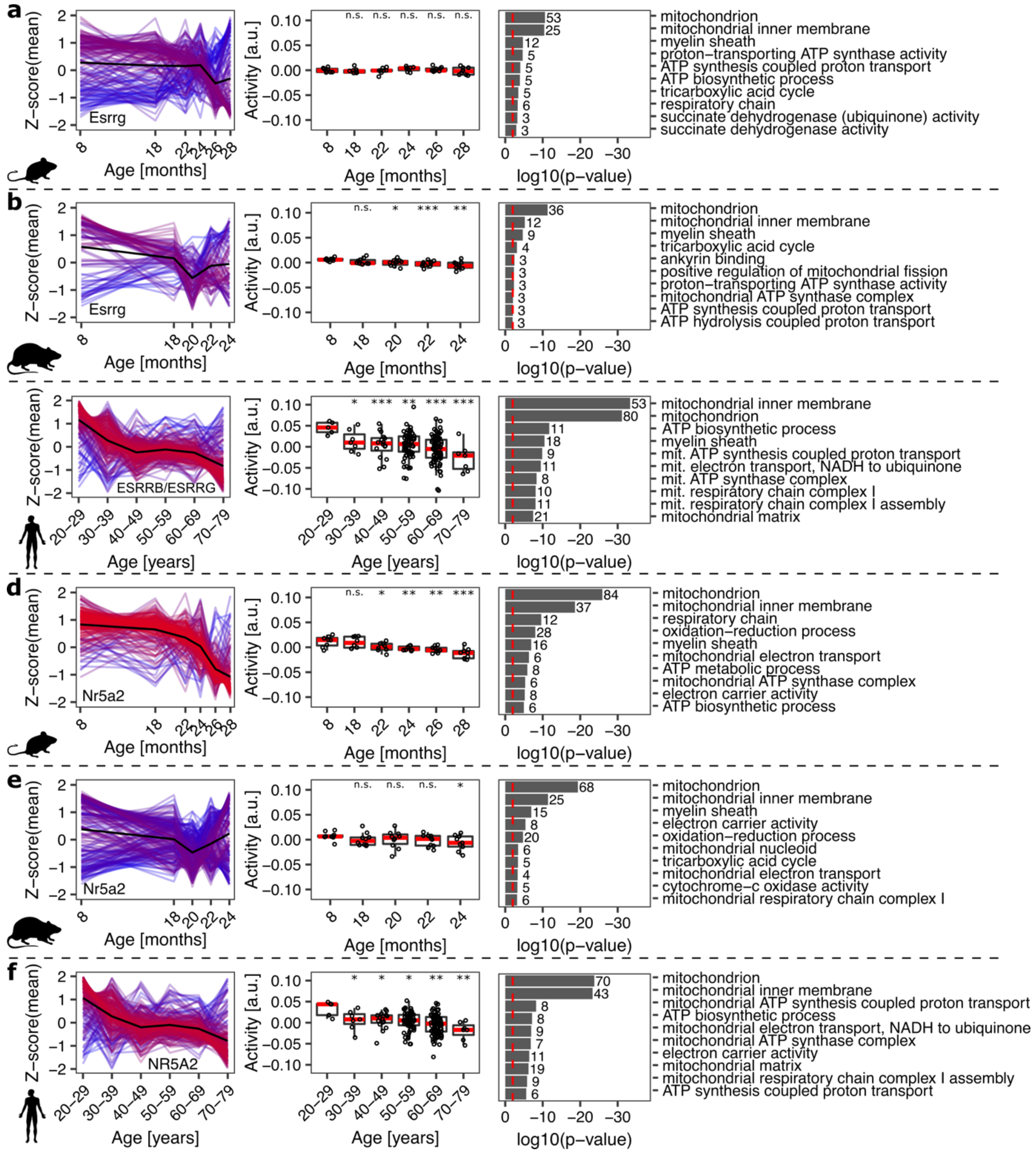

Supplementary Fig. 8. ISMARA-inferred activity of Esrrg, ESRRB/ESRRG and NR5A2 during muscle aging across species. **a-c** Activity of motifs associated with TFs Esrrg, Esrrg and ESRRB/ESRRG, which were predicted to regulate muscle aging-related genes in mouse, rat and human, respectively. **d-f** Similar for the TF Nr5a2 (NR5A2 for humans). The 1st column depicts the normalized expression (z-scores of mean log2(TPMs)) of the top 300 target genes. The mean value per age (or age group) across genes is indicated by the black (reference) line. Gene expression time course lines were colored by the distance from the reference line: red- close to the reference line, blue- far from the reference line. The 2nd column depicts the activity of the corresponding TFs predicted by ISMARA. *, ** and *** denote a significant difference based on two-sided Student's t-test between the youngest age/age group and all other ages/age groups of p-value<0.05, p-value<0.01 and p-value<0.001, respectively; 'n.s.'- not significant (p-value≥0.05). The 3rd column shows the 10 most enriched GO terms among the 300 highest-scoring targets of the TFs. GO analysis was performed in DAVID[87]. Red dashed lines indicate the significance threshold (p-value<0.01). The numbers next to the bars denote how many genes were attributed to an enriched GO term.

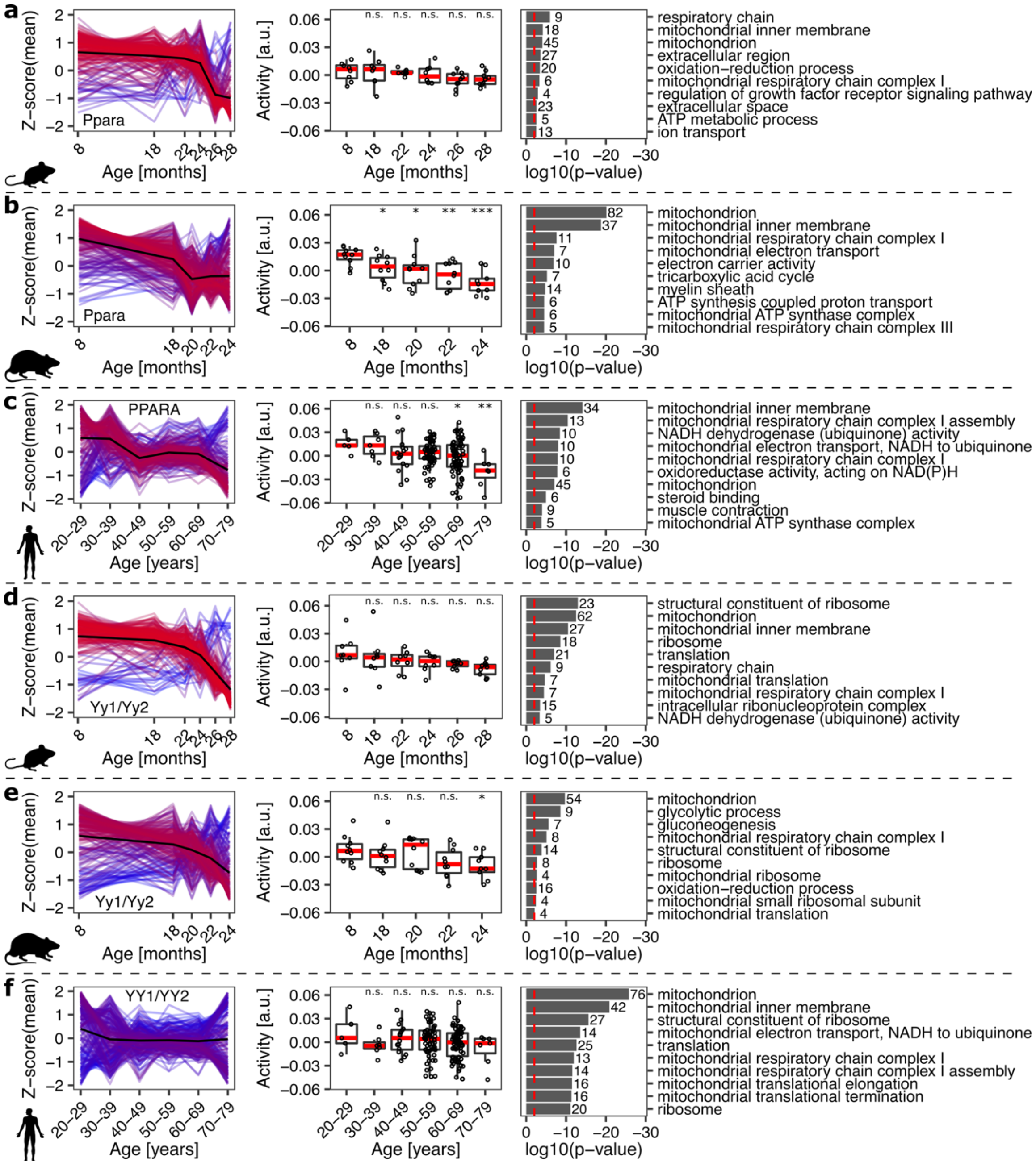

Supplementary Fig. 9. ISMARA-inferred activity of PPARA and YY1/2 during muscle aging across species. **a-c** Activity of the motif associated with the TF Ppara (PPARA for humans), which was predicted to regulate muscle aging-related genes in mouse, rat and human, respectively. **d-f** Similar for the TF Yy1/2 (YY1/2 for humans). The 1st column depicts the normalized expression (z-scores of mean log2(TPMs)) of top 300 target genes. The mean value per age (or age group) across genes is indicated by the black (reference) line. Gene expression time course lines were colored by the distance from the reference line: red- close to the reference line, blue- far from the reference line. The 2nd column depicts the activity of the corresponding TFs predicted by ISMARA. *, ** and *** denote a significant difference based on two-sided Student's t-test between the youngest age/age group and all other ages/age groups of p-value<0.05, p-value<0.01 and p-value<0.001, respectively; 'n.s.'- not significant (p-value≥0.05). The 3rd column shows the 10 most enriched GO terms among the 300 highest-scoring targets of the TFs. GO analysis was performed in DAVID[87]. Red dashed lines indicate the significance threshold (p-value<0.01). The numbers next to the bars denote how many genes were attributed to an enriched GO term.

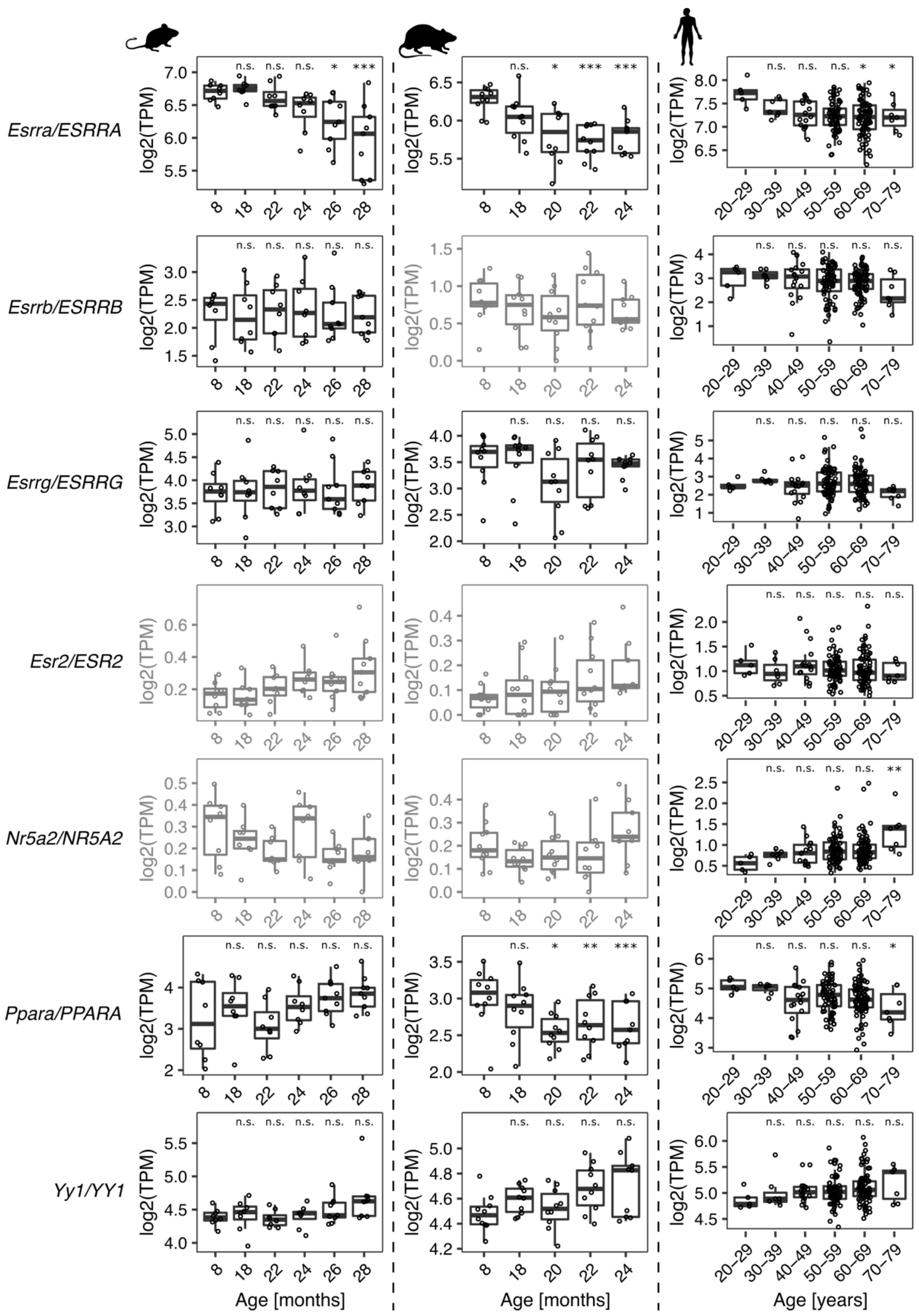

Supplementary Fig. 10. Expression of genes encoding TFs commonly regulated during gastrocnemius muscle aging in mouse, rat and human. *, ** and *** denote a significant difference based on differential expression analysis between the youngest age/age group and all other ages/age groups of FDR<0.05, FDR<0.01 and FDR<0.001, respectively; 'n.s.'- not significant (FDR≥0.05). Lowly expressed genes were excluded from the statistical analysis and their expression plots are shaded.

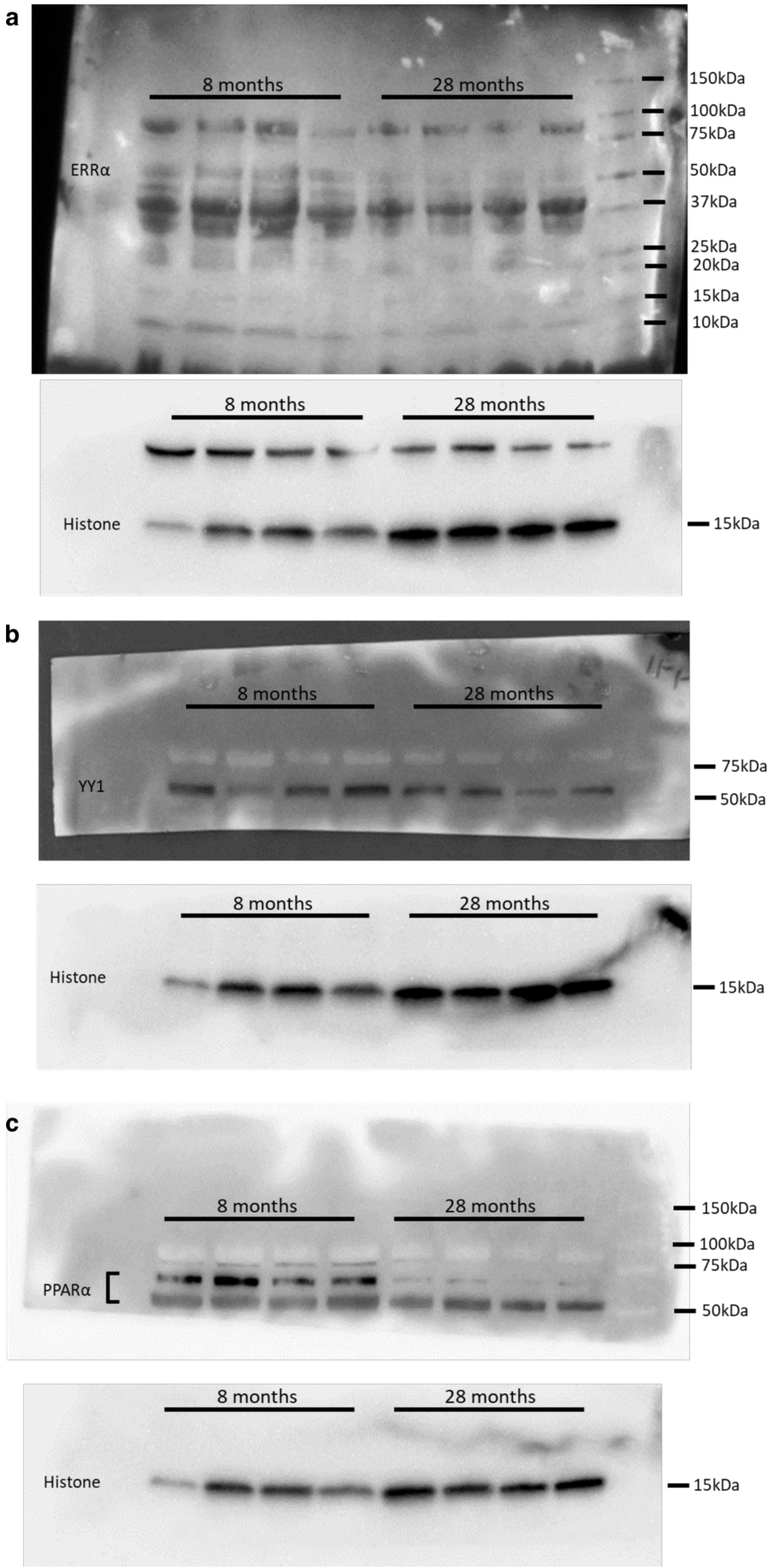

Supplementary Fig. 11. Full uncropped blot images of Fig. 7a. **a** ERRα. **b** YY1. **c** PPARα.

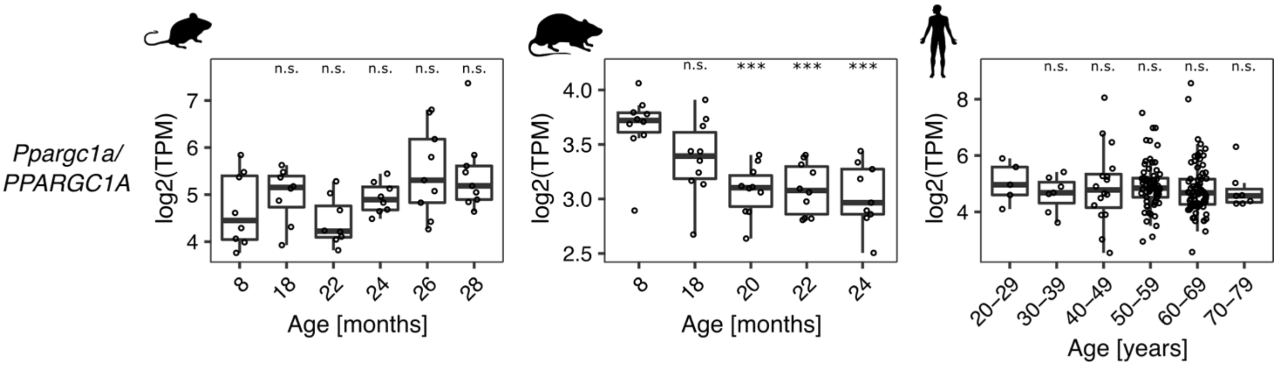

Supplementary Fig. 12. Expression of the gene encoding PGC-1α (*Ppargc1a* in rodents, *PPARGC1A* in human) during gastrocnemius muscle aging in mouse, rat and human. *** denotes a significant difference based on two-sided Student's t-test between the youngest age group and all other age groups of p-value<0.001; 'n.s.'- not significant (p-value≥0.05).

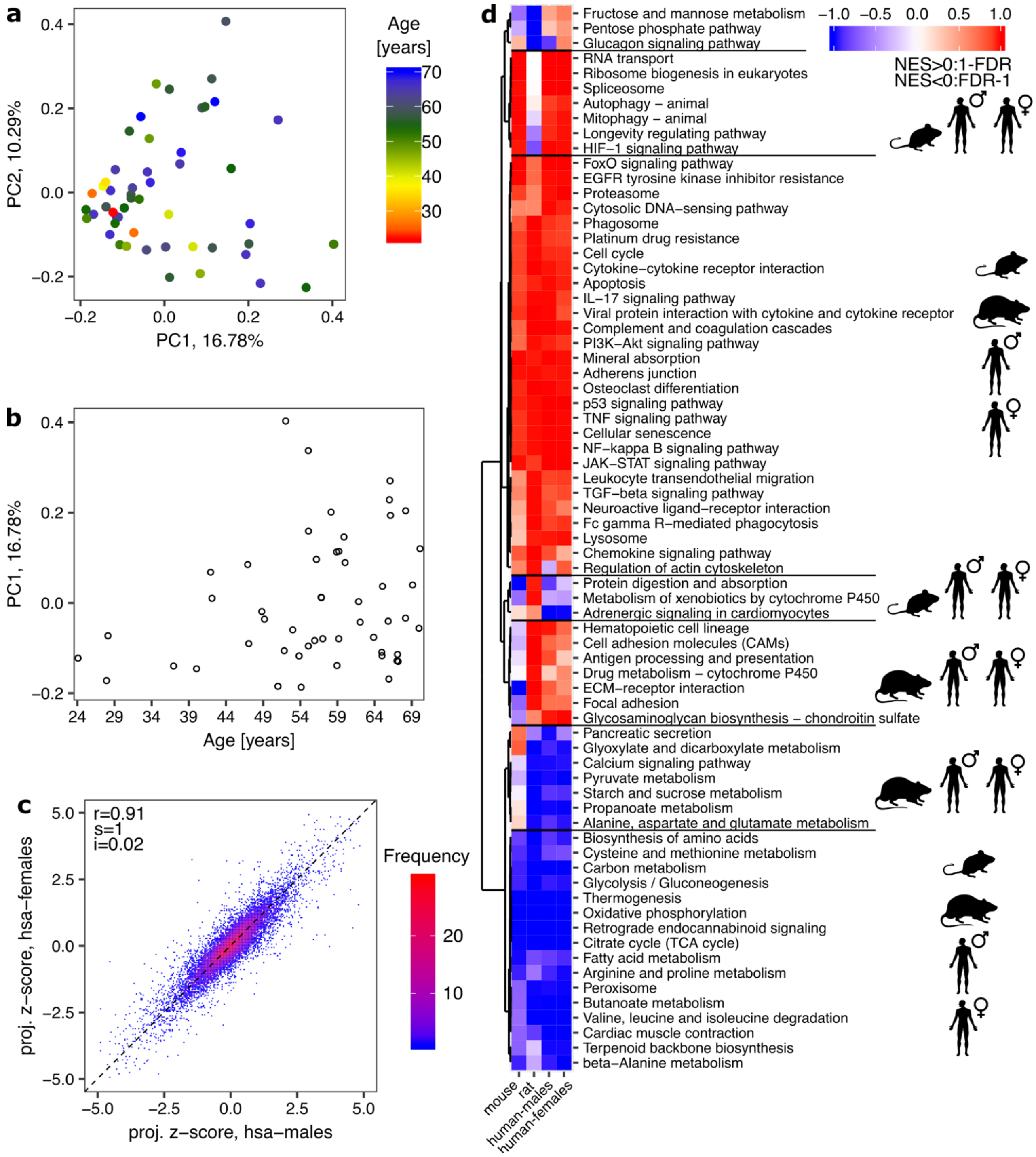

Supplementary Fig. 13. Changes in gene expression and pathway activities during gastrocnemius aging in female humans in comparison to other species. **a** Principal component analysis of transcript abundances during muscle aging in female humans. **b** PC1 coordinates for the RNA-Seq data set collected for female human gastrocnemius muscles. **c** Correlation between the standardized PC1 projections for individual genes (projection z-scores) for female and male humans. 'hsa' designates '*Homo sapiens*' (human). 'r' indicates the value of the Pearson correlation coefficient. Black dashed line corresponds to the direction of the highest variance for the comparison, with the slope 's' and intercept 'i'. **d** Heatmap summarizing the enrichment of KEGG pathways among genes ranked by projection z-scores for mouse, rat and male and female humans, respectively. A pathway was included in the heatmap if it was significantly enriched in at least one organism with the significance threshold FDR<0.01. Hierarchical clustering revealed pathways with a similar response during muscle aging in two or more species.
