# Supplementary material for: Molecular and phenotypic analysis of rodent models reveals conserved and species-specific modulators of human sarcopenia
Source: Commun Biol. 2021 Feb 12;4:194. doi: 10.1038/s42003-021-01723-z (PMC7881157; doi:10.1038/s42003-021-01723-z)
Supplement: Supplementary file 3 — Description of Additional Supplementary Files [file 42003_2021_1723_MOESM3_ESM.pdf]

Description of Additional Supplementary Files

File name: Supplementary Data 1.

Description: Experimental measurements in rodents underlying the graphs and charts of the study.
