# Supplementary material for: Molecular and phenotypic analysis of rodent models reveals conserved and species-specific modulators of human sarcopenia
Source: Commun Biol. 2021 Feb 12;4:194. doi: 10.1038/s42003-021-01723-z (PMC7881157; doi:10.1038/s42003-021-01723-z)
Supplement: Supplementary file 5 — Reporting Summary [file 42003_2021_1723_MOESM5_ESM.pdf]

nature research

Corresponding author(s): Mihaela Zavolan

Last updated by author(s): Dec 18, 2020

# Reporting Summary

Nature Research wishes to improve the reproducibility of the work that we publish. This form provides structure for consistency and transparency in reporting. For further information on Nature Research policies, see our Editorial Policies and the Editorial Policy Checklist.

## Statistics

For all statistical analyses, confirm that the following items are present in the figure legend, table legend, main text, or Methods section.

| n/a | Confirmed | |
|---|---|---|
| ☐ | ☒ | The exact sample size (*n*) for each experimental group/condition, given as a discrete number and unit of measurement |
| ☐ | ☒ | A statement on whether measurements were taken from distinct samples or whether the same sample was measured repeatedly |
| ☐ | ☒ | The statistical test(s) used AND whether they are one- or two-sided<br>*Only common tests should be described solely by name; describe more complex techniques in the Methods section.* |
| ☐ | ☒ | A description of all covariates tested |
| ☐ | ☒ | A description of any assumptions or corrections, such as tests of normality and adjustment for multiple comparisons |
| ☐ | ☒ | A full description of the statistical parameters including central tendency (e.g. means) or other basic estimates (e.g. regression coefficient) AND variation (e.g. standard deviation) or associated estimates of uncertainty (e.g. confidence intervals) |
| ☐ | ☒ | For null hypothesis testing, the test statistic (e.g. *F*, *t*, *r*) with confidence intervals, effect sizes, degrees of freedom and *P* value noted<br>*Give P values as exact values whenever suitable.* |
| ☒ | ☐ | For Bayesian analysis, information on the choice of priors and Markov chain Monte Carlo settings |
| ☐ | ☒ | For hierarchical and complex designs, identification of the appropriate level for tests and full reporting of outcomes |
| ☐ | ☒ | Estimates of effect sizes (e.g. Cohen's *d*, Pearson's *r*), indicating how they were calculated |

*Our web collection on statistics for biologists contains articles on many of the points above.*

## Software and code

Policy information about availability of computer code

| | |
|---|---|
| Data collection | Processing of RNA-Seq data was performed with the software Cutadapt v1.9.1, kallisto v0.43, Snakemake, R version 3.6.0, R packages RDAVIDWebService_1.22.0, GOstats_2.50.0, Category_2.50.0, Matrix_1.2-17, AnnotationDbi_1.46.0, IRanges_2.18.0, S4Vectors_0.22.0, Biobase_2.44.0, graph_1.62.0, BiocGenerics_0.30.0, ggplot2_3.1.1, gridExtra_2.3, edgeR_3.26.1, limma_3.40.0, matrixStats_0.54.0, ggdendro_0.1-20, reshape2_1.4.3, venn_1.7, biomaRt_2.40.0, KEGGREST_1.24.0, Shiny_0.14.2; GSEA v4.0.3 software |
| Data analysis | The source code to replicate the analysis presented in this study is available from Zenodo at http://doi.org/10.5281/zenodo.3757418. |

For manuscripts utilizing custom algorithms or software that are central to the research but not yet described in published literature, software must be made available to editors and reviewers. We strongly encourage code deposition in a community repository (e.g. GitHub). See the Nature Research guidelines for submitting code & software for further information.

## Data

Policy information about availability of data

All manuscripts must include a data availability statement. This statement should provide the following information, where applicable:

- Accession codes, unique identifiers, or web links for publicly available datasets
- A list of figures that have associated raw data
- A description of any restrictions on data availability

RNA-Seq data set describing gastrocnemius aging in male C57BL/6JRj mice was deposited to Gene Expression Omnibus (GEO, https://www.ncbi.nlm.nih.gov/geo/) under the accession number GSE145480. RNA-Seq data set characterizing gastrocnemius aging in male Wistar rats was submitted to GEO under accession numbers GSE78702 and GSE146976 (series GSE146977). Supplementary Table 1 contains phenotypic parameters of animals used for sequencing. For studying gastrocnemius aging in humans we used RNA-Seq data set obtained from the GTEx project (dbGaP accession number phs000424.v8.p2). The phenotype data table including the age of individuals from whom muscle samples were collected and used for sequencing was obtained from the dbGaP annotation file of the GTEx project and has

restricted access.

# Field-specific reporting

Please select the one below that is the best fit for your research. If you are not sure, read the appropriate sections before making your selection.

- [x] Life sciences
- [ ] Behavioural & social sciences
- [ ] Ecological, evolutionary & environmental sciences

For a reference copy of the document with all sections, see nature.com/documents/nr-reporting-summary-flat.pdf

# Life sciences study design

All studies must disclose on these points even when the disclosure is negative.

| | |
|---|---|
| Sample size | For mice, the number of replicates per age was set to n=8 and n=9 for ages 8, 18, 22 and 24 and 26 and 28 months, respectively. For rats, the number of replicates per age was set to n=10 and n=9 for ages 8, 18, 20 and 22 and 24 months, respectively. These numbers are within the standard range of replicate numbers for high-throughput studies corresponding to 5-10 replicates per condition. |
| Data exclusions | There was no sample exclusion for the analysis performed for rodents. For male humans, we used 181 gastrocnemius RNA-Seq samples from distinct individuals aged between 22 and 70 years selected by the following criteria: (i) only samples notified as 'Eligible For Study' were included in the analysis; (ii) samples of only postmortem donors were considered; (iii) to exclude gender bias, we considered samples of only males; (iv) to exclude the bias induced by prolonged illnesses, only samples of individuals with the death classification '1' (violent and fast death) and '2' (fast death of natural causes) based on the 4-point Hardy scale were considered. The same criteria were applied for the selection of gastrocnemius samples of female humans (51 samples from distinct individuals aged between 24 and 70 years). |
| Replication | Replication quality was checked by principal component analysis. |
| Randomization | Rodents were randomly allocated to experimental groups. |
| Blinding | Investigators were blinded to group allocation during data collection and analysis. |

# Reporting for specific materials, systems and methods

We require information from authors about some types of materials, experimental systems and methods used in many studies. Here, indicate whether each material, system or method listed is relevant to your study. If you are not sure if a list item applies to your research, read the appropriate section before selecting a response.

## Materials & experimental systems

| n/a | Involved in the study | |
|---|---|---|
| [x] | [ ] | Antibodies |
| [x] | [ ] | Eukaryotic cell lines |
| [x] | [ ] | Palaeontology and archaeology |
| [ ] | [x] | Animals and other organisms |
| [x] | [ ] | Human research participants |
| [x] | [ ] | Clinical data |
| [x] | [ ] | Dual use research of concern |

## Methods

| n/a | Involved in the study | |
|---|---|---|
| [x] | [ ] | ChIP-seq |
| [x] | [ ] | Flow cytometry |
| [x] | [ ] | MRI-based neuroimaging |

## Animals and other organisms

Policy information about studies involving animals; ARRIVE guidelines recommended for reporting animal research

| | |
|---|---|
| Laboratory animals | Male, C57BL/6JRj mice were purchased from the aging colony at Janvier Labs (Le Genest-Saint-Isle, France). |
| Wild animals | n/a |
| Field-collected samples | n/a |
| Ethics oversight | All mouse related procedures were performed in accordance with Swiss regulations for animal experimentation and approved by the veterinary commission of the Canton Basel-Stadt. |

Note that full information on the approval of the study protocol must also be provided in the manuscript.
